# Robotic assessment of bilateral and unilateral upper limb functions in adults with cerebral palsy

**DOI:** 10.1186/s12984-024-01415-9

**Published:** 2024-08-22

**Authors:** I. Poitras, S. P. Dukelow, A. Campeau-Lecours, C. Mercier

**Affiliations:** 1 Center for Interdisciplinary Research in Rehabilitation and Social Integration, Quebec City, Quebec Canada; 2 School of Rehabilitation Sciences, Laval University, Quebec City, Quebec Canada; 3 Hotchkiss Brain Institute, University of Calgary, Calgary, Alberta Canada; 4 Department of Clinical Neurosciences, University of Calgary, Calgary, Alberta Canada; 5 Department of Mechanical Engineering, Laval University, Quebec City, Quebec Canada

**Keywords:** Cerebral palsy, Reaching, Bimanual coordination, Motor function, Robotic assessment, Adult

## Abstract

**Background:**

Children with unilateral cerebral palsy (CP) exhibit motor impairments predominantly on one side of the body, while also having ipsilesional and bilateral impairments. These impairments are known to persist through adulthood, but their extent have not been described in adults with CP. This study’s aim is to characterize bilateral and unilateral upper limbs impairments in adults with CP.

**Methods:**

Nineteen adults with CP (34.3 years old ± 11.5) performed three robotic assessments in the Kinarm Exoskeleton Lab, including two bilateral tasks (Object Hit [asymmetric independent goals task] and Ball on Bar [symmetric common goal task]) and one unilateral task (Visually Guided Reaching, performed with the more affected arm [MA] and less affected arm [LA]). Individual results were compared to sex, age and handedness matched normative data, describing the proportion of participants exhibiting impairments in each task-specific variable (e.g., Hand speed), each performance category (e.g., Feedforward control) and in global task performance. Associations were assessed using Spearman correlation coefficients between: 1: the results of the MA and LA of each limb in the unilateral task; and 2: the results of each limb in the unilateral vs. the bilateral tasks.

**Results:**

The majority of participants exhibited impairments in bilateral tasks (84%). The bilateral performance categories (i.e., Bimanual) identifying bilateral coordination impairments were impaired in the majority of participants (Object Hit: 57.8%; Ball on Bar: 31.6%). Most of the participants were impaired when performing a unilateral task with their MA arm (63%) and a smaller proportion with their LA arm (31%). The Feedforward control was the unilateral performance category showing the highest proportion of impaired participants while displaying the strongest relationship between the MA and LA arms impairments (r_s_ = 0.93). Feedback control was the unilateral performance category most often associated with impairments in bilateral tasks (6 out of 8 performance categories).

**Conclusions:**

Adults with CP experienced more impairment in bilateral tasks while still having substantial impairments in unilateral tasks. They frequently display Feedforward control impairments combined with a higher reliance on Feedback control during both bilateral and unilateral tasks, leading to poorer motor performance.

**Supplementary Information:**

The online version contains supplementary material available at 10.1186/s12984-024-01415-9.

## Introduction

Assessing impairments in bilateral tasks in neurologically impaired patients is essential to understanding the motor impairments observed in those populations (e.g., stroke, people with cerebral palsy [CP]). A review from Kantak et al. [1] has proposed a classification of bimanual tasks based on two characteristics: (1) the symmetry of arm movements (asymmetric or symmetric task); and (2) the conceptualization of task goal (independent goals or common goal). This helps understand the underlying impairments and mechanisms as asymmetric tasks are harder to execute. Indeed, more variability in movement patterns across trials is observed during these tasks compared to symmetric tasks, and they have been shown to solicit different neural networks [1]. Interestingly, Kim and Kang [2] have shown in their systematic review that stroke survivors are more impaired during bilateral asymmetric tasks than during symmetric tasks. Some studies have also shown that children with CP display impairments during bilateral asymmetric tasks [3, 4] and symmetric tasks [5]. In adults with CP, it has been demonstrated that in bilateral tasks, the dominant arm slows down to match the capacities of the non dominant arm, resulting in temporal and spatial coupling of both hands [6, 7]. This suggests that the characteristics of motor tasks could also impact bilateral performance in adults with CP.

The most prevalent type of CP is the spastic hemiplegic CP in which the individual exhibits upper limb motor impairments predominantly on one side of the body [8, 9] while also displaying milder impairments in the other side of the body [10] as well as bilateral coordination impairments [4, 11]. This is why the sides contralateral and ipsilateral to the lesion are generally respectively called the more affected (MA) and less affected (LA) arm, rather than the affected and unaffected arm. Performance in bilateral tasks might differ depending on whether the sensorimotor impairments are restricted to the side contralateral to the lesion or affect both upper limbs. While a study in children with hemiplegic CP has reported a strong relationship between some assessments of bilateral performance and unilateral capacity [12], the specific impairments interfering the most with bilateral and unilateral tasks might differ, specifically regarding the task characteristics presented above. Indeed, the performance during bilateral tasks depends not only on the individual capacity of each limb, but also on the capacity to coordinate the use of both limbs, and could be influenced by the task complexity (asymmetric vs. symmetric). Moreover, it has been shown that individuals with CP display motor planning impairments during unilateral task [13], but little is known about their impact on bimanual performance. Motor control theory supports a relationship between Feedforward control (i.e., motor planning), Feedback control and unilateral motor performance [14]. Given that most activities of daily living require a coordinated use of the hands, a better understanding of the sensorimotor impairments (e.g., Feedforward control vs. Feedback control) observed during the realization of bilateral (asymmetric independent and symmetric common goal task) and unilateral tasks, and of the association between bilateral and unilateral tasks, is needed.

The general aim of this study was to characterize bilateral and unilateral upper limbs motor impairments in adults with CP. To address the effect of task characteristics, we used two bilateral tasks, an asymmetric task with independent goals (the most complex task) and a symmetric task with a common goal (the simpler task). Moreover, given that the relationship between impairments in bilateral and unilateral tasks is still unclear in adults with CP, we also assessed the unilateral impairments in the MA and LA arms by using a typical unilateral task, the visually guided reaching. Taken together, these assessments allowed for the description of bilateral and unilateral functions while evaluating the relationship between the MA and the LA arms capacities, as well as with the bilateral performance.

The three specific objectives, with their respective hypotheses, are:

1. To compare the frequency of occurrence of various types of impairments observed during bilateral (asymmetric independent goals and symmetric common goal) and unilateral upper limb tasks in adults with CP having mild to moderate motor impairments, and determine whether they differ between levels of impairments as characterized by the Manual Ability Classification System (MACS I to III);
  Hypotheses:
  - Bilateral impairments will be more frequent than unilateral impairments.
  - All the tasks will exhibit at least a moderate association with  the severity of impairments (MACS level).
2. To explore the relationship between the impairments of the MA and LA arm during a unilateral task;
  - A moderate association will be found between impairments of the MA and LA arm (r_s_ > 0.5).
3. To explore the relationship between impairments in bilateral and unilateral tasks observed in each limb;
  - A moderate association will be found between impairments observed in bilateral and unilateral tasks (r_s_ > 0.5), and in particular for Feedforward and Feedback control.

## Methods

### Participants

Participants were recruited through the Université Laval mailing list, the health records of the Centre intégré universitaire de santé et de services sociaux de la Capitale-Nationale (CIUSSS-CN) and through patient organizations. The inclusion criteria were (1) being aged from 18 to 65 years old; (2) having a diagnosis of hemiplegic CP; (3) being able to perform a transfer with minor assistance (to the wheelchair of the robotic device used for assessment); (4) having a level of I, II, or III on the MACS [15]. Exclusion criteria were (1) having cognitive impairments restricting understanding of the task to perform, and (2) having uncorrected visual problems interfering with the assessment tasks. The study was approved by the local ethic committee (Ethics #2018-609, CIUSSS-CN) and all the participants provided their written inform consent prior to the study.

Two clinical assessments allowed characterizing the group: the Jebsen Taylor Hand Function Test (JTHFT) and the MACS. The JTHFT assesses the unilateral upper limb capacities in seven functional tasks. A participant was considered as impaired if their score fell outside of the normative range [16]. The MACS level describes the capacity of individuals with CP to handle objects in everyday activities [15, 17]. There are five levels, but participants in the present study were between level I (objects are handled easily and successfully) to III (objects are handled with difficulty—help is needed to prepare and/or modify activities).

### Robotic assessments

**Fig. 1:**
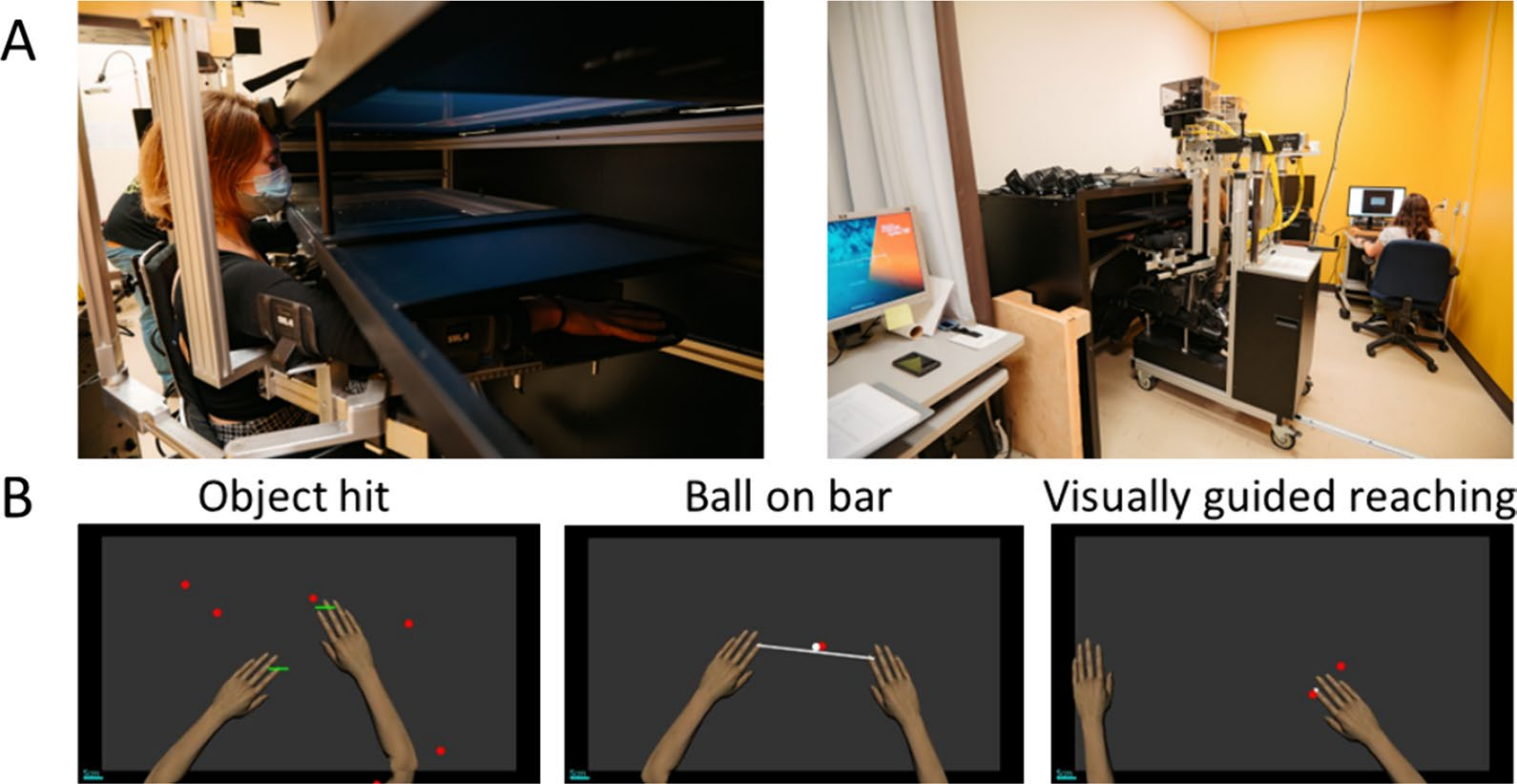
**A** Experimental setup for the Kinarm Exoskeleton Lab. **B** Workspace representation of the three robotic tasks

Participants completed one session, lasting ~ 2 h, comprising several tasks performed with a robotic assessment system, the Kinarm Exoskeleton Lab (Kinarm, Kingston, Ontario). The experimental set up as well as an example of each task are shown in Fig. 1. The participant was seated in a chair with their arms placed in robotic arm troughs. The robotic system permitted movements of the arm in the horizontal plane involving flexion and extension movements at the shoulder and elbow joints and reduced the effects of gravity. A 2D virtual-reality system allowed control over visual stimuli and real-time feedback about arms position. Three tasks have been selected from the standard tasks available from the company and are detailed below. The tasks were always performed in the same order (following the presentation order in the text) after performing the anthropometric adjustments and the calibration. A report comparing the results of the participant to sex, age and handedness matched normative data and a comma-separated values (csv) files were obtained at the end of each session. The instructions given to the participants were those provided by the company, as the tasks were standardized. The csv files provided the z-score for each evaluated task-specific variables and a composite score (*Task score*). The z-score of the task-specific variables were obtained by transforming the raw score using a model of healthy controls [18] (see https://kinarm.com/kinarm-products/kinarm-standard-tests). An individual with CP was considered as impaired on a task-specific variable if their performance fell outside of the 97.5% performance range of healthy controls (z-score < − 1.96 or > 1.96), depending on the side to indicate a poor performance for each task-specific variable—see Table 1 in Supplementary materials for a description of all the task-specific variables).

Task-specific variables were grouped in performance categories based on previous studies [19–21], allowing describing specific sensorimotor control impairments. Given that z-score < − 1.96 or > 1.96 could indicate a bad performance depending on the task-specific variable, the sign was inverted for some variables so that a negative value always reflected a good performance and a positive value a bad performance. After the values were converted, we used the mean value of the task-specific variables by categories to perform the statistical analysis. The performance categories were considered as impaired for an individual if the computed mean z-score fell outside of the normative value.

To quantify the overall performance, a *Task score* was calculated for each participant and each Task (Object Hit, Ball on Bar and Visually Guided Reaching). The *Task score* was calculated by using the value of a set of task-specific variables for each task, and the mathematical process was already described in [18] (for details, see https://kinarm.com/kinarm-products/kinarm-standard-tests). An individual with CP was considered as impaired on their overall performance if their performance fell outside of the 97.5% performance range of healthy control (z-score < − 1.96 or z-score > 1.96 depending on the side to indicate a poor performance for each task-specific variables). See a detailed description of each task and their performance categories below.

#### Object Hit (bilateral asymmetric independent goals task)

Participant used the hand of their choice to hit balls moving from the distal part of the screen to the proximal part (i.e., toward them) in different medial–lateral positions [19]. Three hundred balls were presented at a gradually increasing frequency and speed. The task was performed twice and the analyses were performed on the second attempt to reduce the impact of learning [22]. The task-specific variables calculated from kinematic data were categorized in four performance categories as described by Tyryshkin el al. [19]: (1) Bimanual (*Hand bias hits; Hand speed bias; Movement area bias*), (2) Spatial/temporal (*Miss bias; Hand transition; Median error*); Motor (*Hand speed and Movement area* [MA arm, LA arm]); Global (*number of Targets hit* [total, with the MA arm, with the LA arm]).

#### Ball on Bar (bilateral symmetric common goal task)

In the bilateral Ball on Bar task, a virtual bar was placed between the hands of the subject with a virtual ball on it [20]. Four targets were successively presented to the participant, whose objective was to move the ball into each target as quickly and accurately as possible. The task had three levels of 1 min each. At levels 2 and 3, the ball could “roll” and fall off the bar if tilted, which required precise bilateral control. The level 3 was removed from the analyses as it did not allow for identifying more impaired participants (same results as [20]). The task was performed twice, and the analyses were based on the second attempt to diminish the impact of learning effect [22]. The task-specific variables calculated from kinematic data were categorized in four performance categories as described by Lowrey et al. [20] and [23]: 1: Interlimb (*Hand speed difference; Hand speed peaks bias; Hand path length bias*), 2: Bimanual (*Mean bar tilt; standard deviation of the Bar tilt; Bar length variability*), 3: Hand and ball (*Ball speed; Hand speed* and *Hand speed peaks* [MA arm, LA arm]); 4: Total (*Targets completed; Time to target*).

#### Visually Guided Reaching (unilateral task)

The participant had to point at four targets as quickly and accurately as possible. The targets were spread out over a radius of 10 cm from the starting target and were presented in a pseudo-random order (for a total of 24 reaching movements) [10, 21]. Subject received visual feedback (represented by a white dot) indicating the location of their index finger. This task allowed assessing unilateral voluntary motor control and was performed on the LA arm first, and then with the MA arm. The task-specific variables calculated from kinematic data were categorized in five performance categories as described by Coderre et al. [21]: (1) Postural control (*Posture speed*); (2) Visual reaction (*Reaction time*); (3) Feedforward control (*Initial direction angle and Initial distance ratio*); (4) Feedback control (*Speed maxima count and Min–max speed*); (5) Total movement (*Movement time, Path length ratio, Max speed*).

### Statistical analyses

Descriptive statistics (mean, standard deviation [SD] and range) were computed for sociodemographic and robotic variables. All the variables derived from robotic assessments were converted into z-scores based on the normative data provided by Kinarm (sex, age and handedness matched). The percentage of participants with a performance below the normal range was calculated for each of the task-specific variables and the performance categories. A description of the variables and categories associated with the higher frequency of impairments was provided, as well as a percentage of participants showing impairments in their overall performance (*Task score*) (objective 1). Spearman correlation coefficients were calculated to assess the relationship between the *Task score* of each task and the MACS level (objective 1). To evaluate the relationship between the performance of the MA and the LA arms, Spearman correlation coefficients were computed between the performance categories of the unilateral task (Visually Guided Reaching; objective 2). To assess the relationships between the performance categories of the bilateral (Object Hit and Ball on Bar) and the unilateral (Visually Guided Reaching of the MA arm) tasks, Spearman correlation coefficients were computed (objective 3) [24]. The correlations were described as follows: 0.00–0.09 = negligible, 0.10–0.39 = weak, 0.40–0.69 = moderate, 0.70–0.89 = strong, 0.90–1.00 = very strong [25]. A p-value smaller than 0.05 was considered as significant. Due to the exploratory nature of objectives 2 and 3, no correction for multiple testing was applied for the results presented in the text. Nevertheless, p-values corrected with the Benjamini multiple comparisons correction method are presented in the table for Objective 3 [24], allowing the readers to exert their judgment on the results.

## Results

### Participants

Nineteen participants were recruited for this study. All participants were able to complete the assessments, except one participant who was unable to perform the second trial of the two bilateral robotized tasks due to muscle fatigue and increase in spasticity. The data of the first attempt was used for this participant as Wilcoxon signed-rank test showed no effect of the attempt (first vs. second) in our dataset (p-value > 0.05). Table 1 presents the demographic and clinical characteristics of the sample.

**Table 1:** Demographic and clinical characteristics of the sample

	Total
n	19
Age (years)	34.3 ± 11.5
Sex (Female[F], male[M])	11F,8 M
MORE Affected side (right[R], left [L])	12R, 7L
Number of participants for each level of the Manual Ability Classification System	I = 7 II = 6 III = 6
Results for Jebsen Taylor Hand Function Test (number of participants impaired, mean [range])	MA = 17; 25.9 [1.29 to 128.4] LA = 12; 4.7 [− 0.2 to 33.2]

*MA* more affected, *LA* less affected

### Frequency of impairments observed during bilateral and unilateral upper limb tasks (objective 1)

**Fig. 2:**
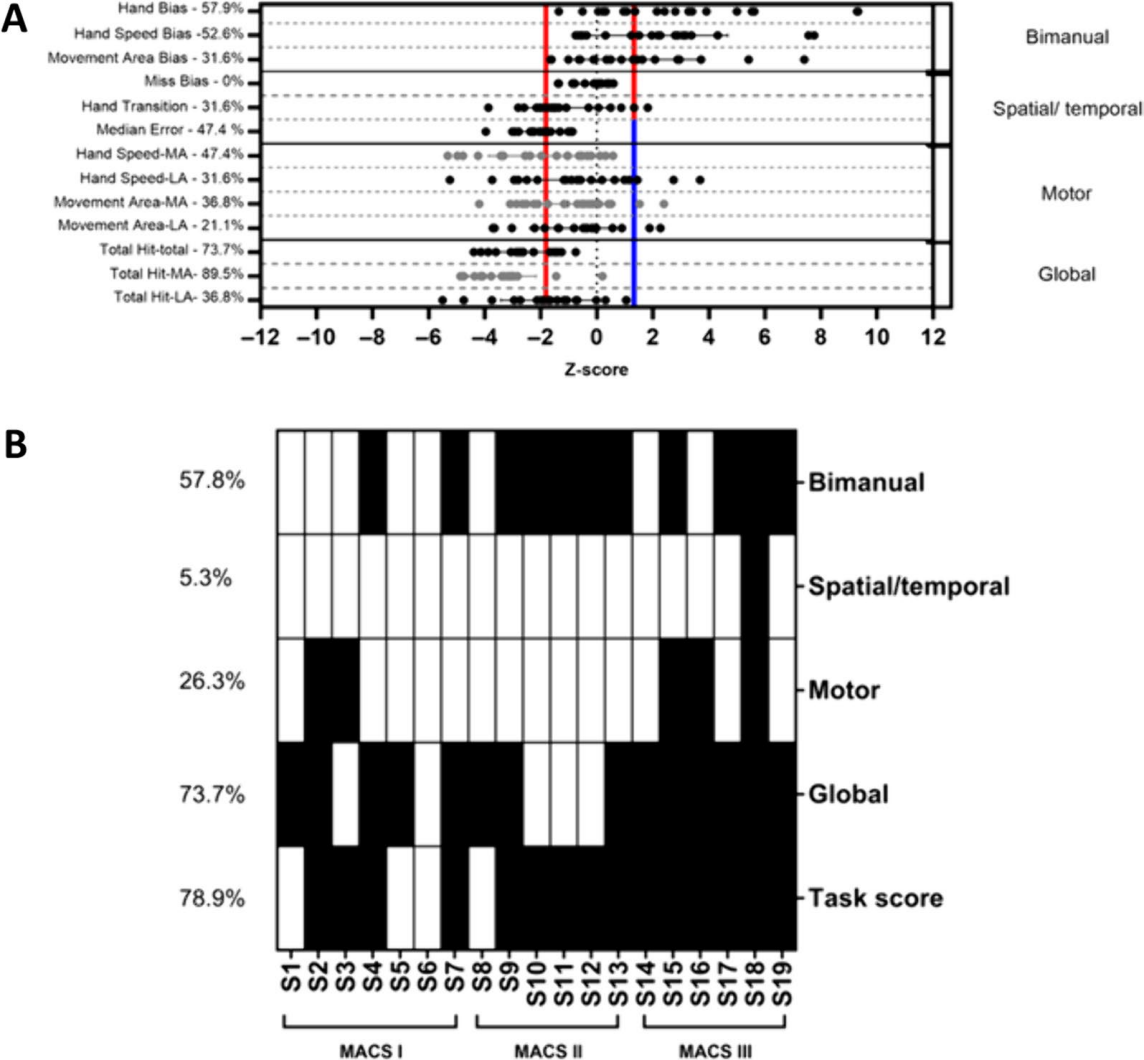
**A** Z-score and standard deviation for each task-specific variable **B** distribution of impairments. **A** Normalized score (Z-score) and standard deviation for each task-specific variable of the Object Hit (bilateral asymmetric independent goals). The grey dots represent the MA arm and the black ones represent LA arm. The red (worse performance than normative data) and blue lines (better performance than normative data) represent the 97.5% performance range of healthy control (z-score = − 1.96 and 1.96). Note that depending on the variable, a positive or negative z-score can represent a deficit. The percentage of participants having impairments for each task-specific variable is indicated. **B** Distribution for each performance categories and across participants. A black square indicating a deficit. The percentage of participants with at least one task-specific variable impaired in each category is indicated. Participants are grouped according to their MACS level; *MA* more affected, *LA* Less affected

**Fig. 3:**
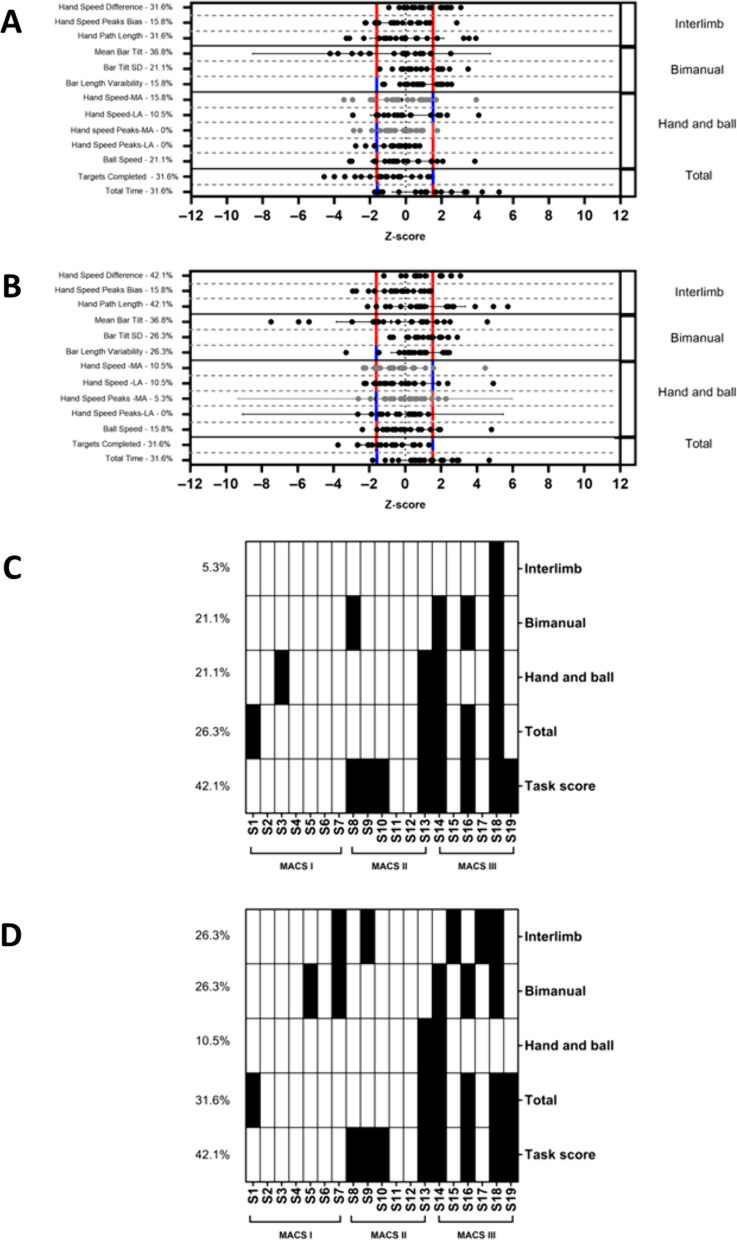
**A**, **B** Z-score and standard deviation for each task-specific variable **C, D** distribution of impairments. **A**, **B** **A** represents the level 1 and **B** the level 2. Normalized score (Z-score) and standard deviation for each variable of the Ball on Bar (bilateral symmetric common goal). The grey dots represent the MA arm and the black ones represent LA arm. The red (worst performance than the norm) and blue lines (better performance than the norm) represent the 97.5% performance range of healthy control (z-score = − 1.96 and 1.96). Note that depending on the variable, a positive or negative z-score can represent a deficit. The percentage of participants having impairments for each task-specific variable is indicated. **C** Distribution of impairments across variables and participants (a black square indicating a deficit). The percentage of impaired participants is indicated for each category. **D** Distribution of impairments across variables and participants (a black square indicating a deficit). The percentage of impaired participants is indicated for each category; *MA* more affected, *LA* less affected

**Fig. 4:**
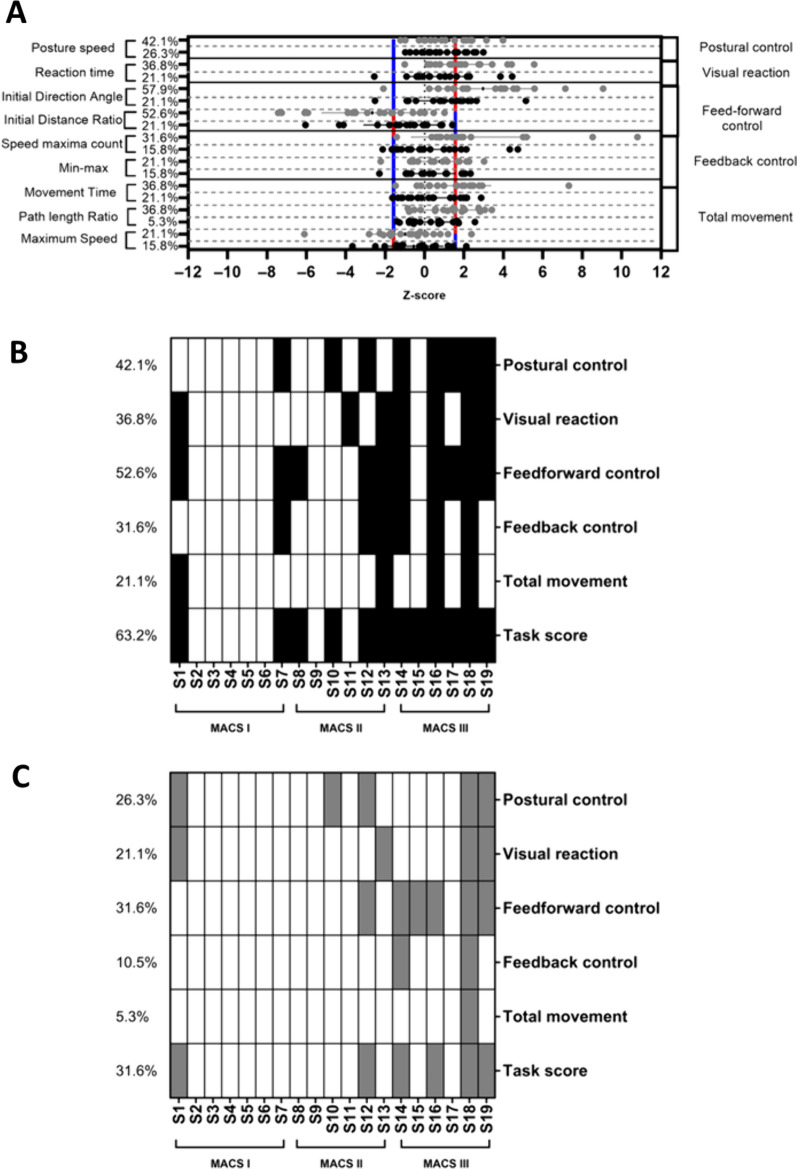
**A** Z-score and standard deviation for each task-specific variable **B**, **C** distribution of impairments. **A** Normalized score (z-score) and standard deviation for each variable of the Visually Guided Reaching for the more affected (MA) arm and the less affected (LA) arm (unilateral). The grey dots represent the MA arm and the black ones represent LA arm. The red (worst performance than the norm) and blue lines (better performance than the norm) represent the 97.5% performance range of healthy controls (z-score = − 1.96 and 1.96). Note that depending on the variable, a positive or negative z-score can represent a deficit. The percentage of participants having impairments for each task-specific variable is indicated. **B** Distribution of impairments across variables and participants for the more affected arm (a black square indicating a deficit). The percentage of impaired participants is indicated for each category. **C** Distribution of impairments across variables and participants for the less affected arm (a grey square indicating a deficit). The percentage of impaired participants is indicated for each category. *MA* more affected, *LA* less affected

Figures 2, 3 and 4 show the mean and standard deviation for each variable and depicts how impairments were distributed across variables and participants for the Object Hit, the Ball on Bar and the Visually Guided Reaching task, respectively.

#### Object Hit (bilateral asymmetric independent goals task)

Participants were outside the range of normal most frequently on the following task-specific variables: *Target hits* (73.7%), the N*umber of target hits* by the MA arm (89.5%), the *Hand bias hits* (57.9%), and the *Hand speed bias* (52.6%) (see Fig. 2A). The Miss bias was the only variable in the normative range for all the participants. The performance categories identified only one participant with no deficit, while the majority of participants had at least impairments in two categories (see Fig. 2B). The performance category with the highest proportion of impaired participants was the *Global* (73.7%, see Fig. 2B). When considering the *Task Score*, 15 participants (78.9%) showed impairments in their overall performance. The *Task score* and the MACS level were moderately associated (r_s_ = 0.51, p-value = 0.03).

#### Ball on Bar (bilateral symmetric common goal task)

For levels 1 and 2 performance on the *Mean bar Tilt* (levels 1 and 2: 36.8%), *Hand speed difference* (level 1: 31.6%, level 2: 42.1%), the *Hand path length* (level 1: 31.6%, level 2: 42.1%), the *Targets completed* (levels 1 and 2: 31.6%), and the *Time to target* (levels 1 and 2: 31.6%) (see Fig. 3A) demonstrated the highest rate of impairment. The other variables showed impairments on the same proportion of participants (10.5 to 26.3%) except for the *Hand speed peak* of the MA and the LA arms (level 1: 0%; level 2: 0 to 5.3%). The task-specific variables identified (at least one variable impaired) seventeen participants with impairments. For the level 1, the number of participants showing impairments in performance categories was evenly distributed across performance categories for the Bimanual, the Hand and ball and the Total with frequency of impairments between 21.1% to 26.3%, with the Interlimb performance category showing a much lower frequency of impairments (i.e., 5.3%). For the level 2, the number of participants showing impairments in performance categories was evenly distributed across performance categories for the Interlimb, the Bimanual and the Total with frequency of impairments between 26.3%, to 31.6%, while the Hand and Ball performance category showing a much lower frequency of impairments (i.e., 10.5%). (see Fig. 3C, D). When considering the *Task Score*, eight participants (42.1%) demonstrated impairments in their overall performance. The *Task score* and the MACS level were strongly associated (r_s_ = 0.75, p-value = 0.002).

#### Visually Guided Reaching (unilateral task)

Participants were outside the range of normal most frequently for the MA arm on the following task-specific variables: the *Initial distance ratio* (52.6%), the *Initial direction angle* (57.9%), and the *Posture speed* (42.1%) (see Fig. 4A). For the LA arm, a limited number of participants demonstrated impairments (5.3 to 26.3% depending on the task-specific variable). Impairments in at least one Task-specific variable were identified in the majority of participants for both the MA arm (68.5%) and the LA arm (47.0%) (see Fig. 4B, C). The performance categories showing the highest rate of impaired participants for the MA arm were the Feedforward control (52.6%) and the Postural control (42.1%). For the LA arm, the performance categories with the highest rate of impairments were the Postural control, the Visual reaction and the Feedforward control (21.1–31.6%). Twelve participants demonstrated a significant impairment on the *Task score* for the MA arm (63.1%) and six participants for the LA arm (31.6%; all of them being also impaired for the MA arm). The *Task score* and the MACS level were moderately associated for both arms (MA: r_s_ = 0.58, p-value = 0.009; LA: r_s_ = 0.54, p-value = 0.02).

To conclude on the results associated to Objective 1, based on the overall *Task score,* the largest number of participants was impaired on the bilateral asymmetric independent goals task (Object Hit) (78.9%). Even participants with mild sensorimotor deficit according to clinical classification (MACS I) were impaired on this task. Performance on this bilateral asymmetric independent goals task was more frequently impaired than that on the unilateral task performed with either the MA arm of the LA arm (63.2% and 31.6% of participants with an impairment, respectively). Performance outside normative values was less frequent for the bilateral symmetric common goal task (Ball on Bar; 42,1%). When looking more specifically at the performance categories to understand the underlying sensorimotor impairments, impairments in Bimanual coordination in the two bilateral tasks were particularly frequent, as were Feedforward control for the MA (and to a lesser extent for the LA) in the unilateral task.

### Relationship between the impairments of the MA and LA arm in the unilateral task (objective 2)

**Fig. 5:**
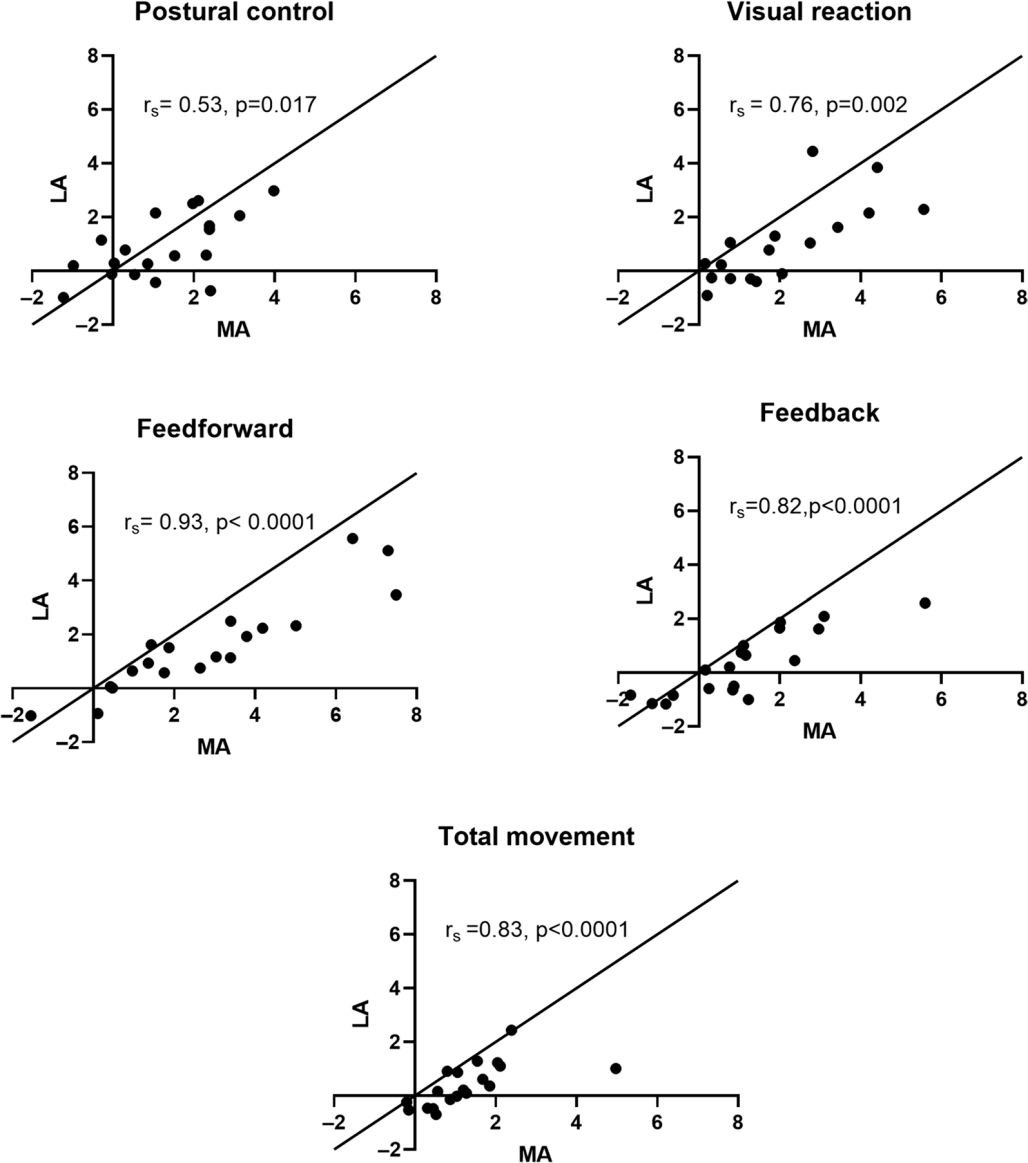
Relationship between the variables calculated for the MA and LA arm in the unilateral task. The identity line allows identifying that even if the LA arm displayed impairments, the impairments were larger in the MA arm (i.e., most points fall below the identity line)

A positive relationship was found between the performance categories of the MA and the LA arms in the Visually Guided Reaching task (see Fig. 5). Notably, a very strong association was for the Feedforward control, although it was not outside of normative values for the LA arm as often as for the MA arm (see previous section). Strong associations were also observed for the Feedback control, the Visual reaction, and the Total movement and a moderate association for the Postural control (all p < 0.05).

### Relationships between impairments in bilateral and unilateral tasks observed in each limb and impairments (objective 3)

Table 2 reports the correlations between the z-scores of the performance categories of the MA arm in the unilateral task (Visually Guided Reaching) and of the performance categories in the two bilateral tasks (Object Hit and Ball on Bar—level 1). The Feedback control of the MA was the category most frequently associated with performance in bilateral tasks, showing a moderate association with 6 out of 8 performance categories in these tasks (r_s_ = 0.43 to 0.65). Postural control and Feedforward control respectively showed moderate to strong association with 4 and 5 performance categories (r_s_ = 0.44 to 0.65). A larger number of significant associations were found between the performance categories of the Visually Guided Reaching and those of the Ball on Bar task (11/20 pairs of variables r_s_ = 0.43 to 0.80) than with those Object Hit (9/20; (r_s_ = 0.40 to 0.57).

**Table 2:** Correlations between the Visually Guided Reaching (more affected arm) and the two bilateral tasks

	Object Hit				Ball on Bar			
	Bimanual	Spatial/temporal	Motor	Global	Interlimb	Bimanual	Hand and ball	Total
Postural control	0.36	0.55**	− 0.14	0.46*	0.58**	0.62**	0.18	0.37
Visual reaction	0.26	0.33	0.09	0.40	0.13	0.34	0.21	0.68**
Feedforward control	0.35	0.45*	0.04	0.57**	0.39	0.65**	0.44*	0.44*
Feedback control	0.49*	0.52**	− 0.04	0.40*	0.48*	0.65**	0.43*	0.31
Total movement	0.13	0.11	0.53**	0.51*	-0.08	0.13	0.51*	0.80**

Significant results are presented in bold. Results for the Object Hit are presented in grey. **Significant results for both the corrected and the uncorrected p-value, *Significant results only for the uncorrected p-value

## Discussion

The general aim of this study was to characterize bilateral and unilateral upper limbs motor impairments in adults with CP. The asymmetric independent goals task (Object Hit) task was the most sensitive one to detect sensorimotor impairments in this population, even in people with a milder CP (MACS I). While several performance categories of the MA arm unilateral task were associated with the global performance in the Object Hit task, few associations were found with other performance categories for that task. Conversely, the symmetric common goal task (Ball on Bar) was less sensitive to the presence of sensorimotor impairments, especially in people with a milder CP, but specific impairments in that task tended to be more often associated with impairments of the MA arm during the unilateral task. All the performance categories of the unilateral task showed an association between the performance of the MA and the LA arms, with a particularly close association for feedforward control.

The vast majority of participants (84%) were impaired for at least one of the two bilateral assessments in the robotic device, vs. 63.2% for at least one arm in the unilateral assessment. Importantly, there was a great difference between the proportion of participants having an abnormal performance in each of the bilateral tasks based on overall *Task score* (Object Hit: 78.9%, Ball on Bar: 42.1%). The difference between the proportion of participants identified as impaired in the three different tasks can potentially be explained by the cognitive demand of the tasks. The asymmetric independent goals task (Object Hit), for which the largest number of participants exhibited impairments, was the task with the highest cognitive demand. It required a high level of visuospatial attention that could explain the impairments observed during the task, especially considering that visuospatial impairments have been frequently reported in children with CP [26]. During this task, the participant must make quick decisions with numerous visual stimuli presented at the same time. This simultaneously challenges the visual system (i.e., rapid processing of visual inputs), the prefrontal cortex (i.e., decision-making process) and the motor system (i.e., coordinated control of movements of the eyes and arms). The two other tasks had more similar cognitive demands as they were required to process one static target at the time. This could also explain why there are more relationships found between the unilateral task and the Ball on Bar task than between the unilateral task and the Object Hit task. The higher number of associations between unilateral impairments and the Ball on Bar task is consistent with similar results reported in children with CP when compared to the Object Hit task [27]. However, most activities of daily living require more cognitive functions than an isolated reaching task toward static targets, suggesting that the relationship between Object Hit task performance and the relative use of the MA arm during activity of daily living should be explored.

An alternative explanation to account for the difference between the two bilateral tasks was the fact that one was asymmetric while the other was symmetric. As mentioned in the Introduction, asymmetric tasks have been reported to be more challenging, especially in individuals with a cerebral lesion (either children with CP or adult stroke survivors). This could be explained by a potentially disrupted excitatory and inhibitory balance between both primary motor cortices during bimanual coordination in adult with CP. For instance, during the execution of a symmetric task, the results of an fMRI study suggested that cortical-level neural crosstalk allows for the stabilization of the movement by providing the same information to both hands [28]. This mechanism makes the interactions during symmetric movements more cost-efficient, but adds complexity to the interactions during asymmetric movements [29]. Indeed, during asymmetric movements, the motor command arriving from the dominant side (LA arm for adults with CP) interferes with the command for the non-dominant side (MA arm for adults with CP), requiring transcallosal inhibition to perform different movements with each hand. These interhemispheric interactions are conveyed through the corpus callosum [30], which have been shown damaged in children with CP [31] and to deteriorate with aging [32]. However, an important methodological aspect is that previous studies investigating the neural substrates of the stability of bimanual movement focused on movements for which homologous muscles were recruited (i.e., mirror-symmetric, compared to parallel out-of-phase movements) rather than on movements in which end-effector move in the same direction (which is the case in the Ball on Bar task, our symmetric task). To the best of our knowledge, no study has compared tasks matching the direction of movement vs. the muscle activation pattern (i.e., co-activation of homologous muscles). Given that overall activity in the motor cortex is tuned to the direction of movement in space [33], it could be hypothesized that matching the movement direction for both limbs should also minimize cortical-level crosstalk, and could potentially induce priming effects similar to those that have been described with mirror-symmetric bimanual movement [34]. This would be an interesting question to address experimentally, especially given that many everyday actions involving manutention of objects requires this type of coordination (e.g., carrying a tray or a box). Overall, these results demonstrated the importance of assessing bilateral functions with different task requirements, as suggested by Kantak et al. [1], in adults with CP to better understand their sensorimotor impairments.

In the unilateral assessment (Visually Guided Reaching), the majority of participants exhibited Feedforward control impairments in their MA (53%), while a smaller proportion (32%) also had such impairments in their LA. The proportion of participants exhibiting Feedback control impairments was also substantial (11 to 32%). Previous studies using the same tasks in children with CP or adult stroke survivors also highlighted Feedforward and Feedback control impairments [10, 21]. The Feedback control was the performance category the more frequently associated with bilateral performance categories while the Feedforward control category was the performance category with the strongest association between MA and LA. This suggests the presence of general impairments in motor planning that impacted movements of both arms and affected bilateral and unilateral performance in a similar way. These impairments in motor planning resulted in a greater reliance on sensory feedback to achieve good performance, which was observed through abnormal scores on the Feedback control performance category. This increased reliance on sensory feedback control to compensate implied that preserved visual and somatosensory function was needed to achieve good performance. However, people with CP display impairments in motor planning [13] as well as visual [35] and somatosensory impairments [36], suggesting that these impairments contributed to the decrease in motor performance.

## Study limitations

The main limitation of this study was the sample size (n = 19), especially given that the population of adults with CP is known as heterogenous. Nevertheless, our sample covered mild to moderate motor impairments with a balanced number of participants across each of the three eligible MACS levels. These results should not be extrapolated to MACS levels IV and V, as the pattern of impairment might differ according to the severity. Moreover, because of the limited sample size and exploratory nature of objective 3, results presented in the text were based on uncorrected p-values for correlation coefficients. Note that corrected p-values are also reported in Table 2, therefore allowing the reader to use their own judgement on the results. A second limitation was that the robotic assessments performed were not fully representative of a real-life context (arms supported against gravity; 2D movements [21, 37]). More ecological assessments (e.g., using accelerometers) are needed to conclude on relative use of the MA during a variety of activities of daily living). The third limitation of this study was the fact that we did not have access to a brain scan for our participants. Therefore, it is not possible to conclude whether the results were similar when grouped by lesion site as demonstrated by [10] in children.

## Conclusion

In conclusion, a vast majority of adults with mild to moderate CP exhibited upper limb motor impairments in a bilateral asymmetric task with high visuospatial requirements. A still large proportion exhibited impairments in bilateral symmetric independent goals task and unilateral tasks with lower visuospatial demand. The presence of impairments in Feedforward control and increased reliance on Feedback control appeared to be key components of both impairments during bilateral and unilateral tasks. The impairments in Feedforward control were strongly correlated between both arms, and motor planning impairments probably contributed substantially to the motor impairments observed in the LA arm. Further studies should focus on understanding the mechanisms underlying motor impairments in bilateral and unilateral tasks for adults with CP as well as the contribution of motor planning of movements and sensory impairments to the upper limbs performance.

### Supplementary Information

Supplementary Material 1.

## Data Availability

The datasets used and/or analysed during the current study are available from the corresponding author on reasonable request.

## Abbreviations

CIUSSS-CN: Centre intégré universitaire de santé et de services sociaux de la Capitale-Nationale
CP: Cerebral palsy
F: Female
JTHFT: Jebsen Taylor Hand Function Test
LA: Less affected
M: Male
MA: More affected
MACS: Manual Ability Classification System

## References

[1] Kantak S, Jax S, Wittenberg G. Bimanual coordination: a missing piece of arm rehabilitation after stroke. Restor Neurol Neurosci. 2017;35(4):347–64. 28697575 10.3233/RNN-170737

[2] Kim RK, Kang N. Bimanual coordination functions between paretic and nonparetic arms: a systematic review and meta-analysis. J Stroke Cerebrovasc Dis. 2020;29(2): 104544. 31818684 10.1016/j.jstrokecerebrovasdis.2019.104544

[3] Hung Y-C, Charles J, Gordon AM. Influence of accuracy constraints on bimanual coordination during a goal-directed task in children with hemiplegic cerebral palsy. Exp Brain Res. 2010;201(3):421–8. 19851759 10.1007/s00221-009-2049-1

[4] Hung Y-C, Charles J, Gordon AM. Bimanual coordination during a goal-directed task in children with hemiplegic cerebral palsy. Dev Med Child Neurol. 2004;46(11):746–53. 15540635 10.1111/j.1469-8749.2004.tb00994.x

[5] Hung Y-C, Spingarn A. Whole body organization during a symmetric bimanual pick up task for children with unilateral cerebral palsy. Gait Posture. 2018;64:38–42. 29843118 10.1016/j.gaitpost.2018.05.028

[6] Langan J, Doyle ST, Hurvitz EA, Brown SH. Influence of task on interlimb coordination in adults with cerebral palsy. Arch Phys Med Rehabil. 2010;91(10):1571–6. 20875516 10.1016/j.apmr.2010.07.015 PMC4005393

[7] Lott C, Johnson MJ. Upper limb kinematics of adults with cerebral palsy on bilateral functional tasks. In: Annual International Conference of the IEEE Engineering in Medicine and Biology Society IEEE Engineering in Medicine and Biology Society Annual International Conference. 2016;2016:5676–9. 10.1109/EMBC.2016.7592015 PMC11774504 28269543

[8] Odding E, Roebroeck ME, Stam HJ. The epidemiology of cerebral palsy: incidence, impairments and risk factors. Disabil Rehabil. 2006;28(4):183–91. 16467053 10.1080/09638280500158422

[9] Himmelmann K, Hagberg G, Uvebrant P. The changing panorama of cerebral palsy in Sweden. X. Prevalence and origin in the birth-year period 1999–2002. Acta Paediatr. 2010;99(9):1337–43. 20377538 10.1111/j.1651-2227.2010.01819.x

[10] Kuczynski AM, Kirton A, Semrau JA, Dukelow SP. Bilateral reaching deficits after unilateral perinatal ischemic stroke: a population-based case-control study. J Neuroengineering Rehabil. 2018;15(1):77. 10.1186/s12984-018-0420-9 PMC6097295 30115093

[11] Wang TN, Howe TH, Liang KJ, Chang TW, Shieh JY, Chen HL. Bimanual motor performance in everyday life activities of children with hemiplegic cerebral palsy. Eur J Phys Rehabil Med. 2021;57(4):568–76. 33733719 10.23736/S1973-9087.21.06504-7

[12] Sakzewski L, Ziviani J, Boyd R. The relationship between unimanual capacity and bimanual performance in children with congenital hemiplegia. Dev Med Child Neurol. 2010;52(9):811–6. 20132142 10.1111/j.1469-8749.2009.03588.x

[13] Martinie O, Mercier C, Gordon AM, Robert MT. Upper limb motor planning in individuals with cerebral palsy aged between 3 and 21 years old: a systematic review. Brain Sci. 2021;11(7):920. 34356154 10.3390/brainsci11070920 PMC8306670

[14] Kawato M. Internal models for motor control and trajectory planning. Curr Opin Neurobiol. 1999;9(6):718–27. 10607637 10.1016/S0959-4388(99)00028-8

[15] Eliasson AC, Krumlinde-Sundholm L, Rösblad B, Beckung E, Arner M, Ohrvall AM, et al. The Manual Ability Classification System (MACS) for children with cerebral palsy: scale development and evidence of validity and reliability. Dev Med Child Neurol. 2006;48(7):549–54. 16780622 10.1017/S0012162206001162

[16] Jebsen RH, Taylor N, Trieschmann RB, Trotter MJ, Howard LA. An objective and standardized test of hand function. Arch Phys Med Rehabil. 1969;50(6):311–9. 5788487

[17] van Meeteren J, Nieuwenhuijsen C, de Grund A, Stam HJ, Roebroeck ME. Using the manual ability classification system in young adults with cerebral palsy and normal intelligence. Disabil Rehabil. 2010;32(23):1885–93. 20450460 10.3109/09638281003611011

[18] Simmatis LER, Early S, Moore KD, Appaqaq S, Scott SH. Statistical measures of motor, sensory and cognitive performance across repeated robot-based testing. J Neuroeng Rehabil. 2020;17(1):86. 32615979 10.1186/s12984-020-00713-2 PMC7331240

[19] Tyryshkin K, Coderre AM, Glasgow JI, Herter TM, Bagg SD, Dukelow SP, et al. A robotic object hitting task to quantify sensorimotor impairments in participants with stroke. J Neuroeng Rehabil. 2014;11:47. 24693877 10.1186/1743-0003-11-47 PMC3992166

[20] Lowrey C, Jackson C, Bagg S, Dukelow S, Scott S. A novel robotic task for assessing impairments in bimanual coordination post-stroke. Int J Phys Med Rehabil. 2014;S3:002. 10.4172/2329-9096.S3-002

[21] Coderre AM, Amr Abou Z, Dukelow SP, Demmer MJ, Moore KD, Demers MJ, et al. Assessment of upper-limb sensorimotor function of subacute stroke patients using visually guided reaching. Neurorehabil Neural Repair. 2010;24(6):528–41. 20233965 10.1177/1545968309356091

[22] Mang CS, Whitten TA, Cosh MS, Scott SH, Wiley JP, Debert CT, et al. Test–retest reliability of the KINARM end-point robot for assessment of sensory, motor and neurocognitive function in young adult athletes. PLoS ONE. 2018;13(4): e0196205. 29689075 10.1371/journal.pone.0196205 PMC5915777

[23] Kinarm Standard Tests Summary (Collection version 3.8.1, analysis version 3.8.1). Kingston, Ontario, Canada.

[24] Yoav B, Daniel Y. The control of the false discovery rate in multiple testing under dependency. Ann Stat. 2001;29(4):1165–88.

[25] Schober P, Boer C, Schwarte LA. Correlation coefficients: appropriate use and interpretation. Anest Analg. 2018;126(5):1763. 10.1213/ANE.0000000000002864 29481436

[26] Ickx G, Hatem SM, Riquelme I, Friel KM, Henne C, Araneda R, et al. Impairments of visuospatial attention in children with unilateral spastic cerebral palsy. Neural Plast. 2018;2018:1435808. 30647728 10.1155/2018/1435808 PMC6311787

[27] Decraene L, Orban de Xivry J-J, Kleeren L, Crotti M, Verheyden G, Ortibus E, et al. In-depth quantification of bimanual coordination using the Kinarm exoskeleton robot in children with unilateral cerebral palsy. J NeuroEng Rehabil. 2023;20(1):154. 37951867 10.1186/s12984-023-01278-6 PMC10640737

[28] Maki Y, Wong KF, Sugiura M, Ozaki T, Sadato N. Asymmetric control mechanisms of bimanual coordination: an application of directed connectivity analysis to kinematic and functional MRI data. Neuroimage. 2008;42(4):1295–304. 18674627 10.1016/j.neuroimage.2008.06.045

[29] Aramaki Y, Osu R, Sadato N. Resource-demanding versus cost-effective bimanual interaction in the brain. Exp Brain Res. 2010;203(2):407–18. 20419370 10.1007/s00221-010-2244-0

[30] Gooijers J, Swinnen SP. Interactions between brain structure and behavior: the corpus callosum and bimanual coordination. Neurosci Biobehav Rev. 2014;43:1–19. 24661987 10.1016/j.neubiorev.2014.03.008

[31] Hung Y-C, Robert MT, Friel KM, Gordon AM. Relationship between integrity of the corpus callosum and bimanual coordination in children with unilateral spastic cerebral palsy. Front Hum Neurosci. 2019;13:334. 31607881 10.3389/fnhum.2019.00334 PMC6769084

[32] Seidler RD, Bernard JA, Burutolu TB, Fling BW, Gordon MT, Gwin JT, et al. Motor control and aging: links to age-related brain structural, functional, and biochemical effects. Neurosci Biobehav Rev. 2010;34(5):721–33. 19850077 10.1016/j.neubiorev.2009.10.005 PMC2838968

[33] Georgopoulos AP, Carpenter AF. Coding of movements in the motor cortex. Curr Opin Neurobiol. 2015;33:34–9. 25646932 10.1016/j.conb.2015.01.012

[34] Byblow WD, Stinear CM, Smith MC, Bjerre L, Flaskager BK, McCambridge AB. Mirror symmetric bimanual movement priming can increase corticomotor excitability and enhance motor learning. PLoS ONE. 2012;7(3): e33882. 22457799 10.1371/journal.pone.0033882 PMC3310871

[35] Ghasia F, Brunstrom J, Gordon M, Tychsen L. Frequency and severity of visual sensory and motor deficits in children with cerebral palsy: gross motor function classification scale. Invest Ophthalmol Vis Sci. 2008;49(2):572–80. 18235001 10.1167/iovs.07-0525

[36] Brun C, Traverse É, Granger É, Mercier C. Somatosensory deficits and neural correlates in cerebral palsy: a scoping review. Dev Med Child Neurol. 2021;63(12):1382–93. 34145582 10.1111/dmcn.14963 PMC9290873

[37] Dukelow SP, Herter TM, Moore KD, Demers MJ, Glasgow JI, Bagg SD, et al. Quantitative assessment of limb position sense following stroke. Neurorehabil Neural Repair. 2010;24(2):178–87. 19794134 10.1177/1545968309345267

