# Supplementary material for: Robotic assessment of bilateral and unilateral upper limb functions in adults with cerebral palsy
Source: J Neuroeng Rehabil. 2024 Aug 22;21:144. doi: 10.1186/s12984-024-01415-9 (PMC11340066; doi:10.1186/s12984-024-01415-9)
Supplement: Supplementary file 1 — Supplementary Material 1. [file 12984_2024_1415_MOESM1_ESM.docx]

Supplementary Materials

Table 1 : Descriptions of the variables measured for each robotic assessment

| Tasks | Task-specific variables | Performance categories | Definition |
| --- | --- | --- | --- |
| Object Hit (15) | Hand bias hits | Bimanual | Value between -1 and 1 identifying which hand is used the most during the task |
|  | Hand speed bias |  | Value between -1 and 1 representing the bias between the hand speed of both hands |
|  | Movement area bias |  | Value from -1 and 1 representing the bias between the movement area of both hands |
|  | Miss bias | Spatial/temporal | Value in centimeter of a bias of misses toward one side of the workspace |
|  | Hand transition |  | Value in square centimeter showing where in the workspace the subject switched their hand preference |
|  | Median error |  | Percentage of the task performed when the participant did half of his errors |
|  | Hand speed of the more affected arm/ less affected arm | Motor | Mean of the hand speed during the task |
|  | Movement area of the more affected arm/ less affected arm |  | Area of the workspace the subject covers during the task |
|  | Target hits | Global | Percentage of the balls hit during the whole task |
|  | Hits more affected arm |  | Percentage of the balls hit during the task with the more affected hand |
|  | Hits less affected arm |  | Percentage of the balls hit during the task with the less affected hand |
| Ball on bar(16, 19) | Targets complete | Total | Number of targets reached with the balls |
|  | Time to target | Hand and ball | Mean time to reach the target |
|  | Ball speed |  | Mean ball speed during the trial |
|  | Hand speed of the more affected arm |  | Mean hand speed of the more affected arm during the trial |
|  | Hand speed of the less affected arm |  | Mean hand speed of the less affected arm during the trial |
|  | Hand speed peak of the more affected arm |  | Number of the speed maxima for the more affected arm |
|  | Hand speed peak of the less affected arm |  | Number of the speed maxima for the less affected arm |
|  | Mean bar tilt | Bimanual | Mean angle of the bar |
|  | Bar tilt standard deviation |  | Standard deviation of the bar angle |
|  | Bar length variability |  | Coefficient of variation of the bar length |
|  | Hand speed difference | Interlimb | Normalized difference in hand speed |
|  | Hand speed peaks bias |  | Relative bias of hand speed peaks |
|  | Hand path length bias |  | Relative bias of hand path length |
| Visually guided reaching(17, 19) | Posture speed | Posture control | Median of the hand speed while the participant waits for the illumination of the peripheral target |
|  | Reaction time | Visual reaction | Time elapsed between the illumination of the peripheral target and the beginning of the movement onset |
|  | Initial direction angle | Feedforward control | Median of the angular deviation between 1) a straight line from the hand position at the movement onset to the hand position after the initial phase of movement and 2) a straight line from the hand position at the movement onset to the end target |
|  | Initial distance ratio |  | Median of the ratio of 1) the distance travelled by the hand during the initial movement phase to 2) the distance travelled between the movement onset and movement offset |
|  | Speed maxima count | Feedback control | Number of maximum hand speeds between the beginning and the end of the movements |
|  | Min-Max speed |  | Mean value of the difference between adjacent minimum and maximum hand speed. |
|  | Movement time | Total movement | Median of the time elapsed from the beginning and the end of the movement. |
|  | Path length ratio |  | Mean of the ratio of the 1) distance travelled by the hand between the movement onset and the movement offset and 2) the straight line between those two positions |
|  | Max speed |  | Median of the maximum speed value between the beginning and the end of the movement |
